# Non-compacted cardiomyopathy: clinical-echocardiographic study

**DOI:** 10.1186/1476-7120-4-35

**Published:** 2006-09-26

**Authors:** Nilda Espinola-Zavaleta, M Elena Soto, Luis Muñóz Castellanos, Silvio Játiva-Chávez, Candace Keirns

**Affiliations:** 1 Echocardiography in Outpatient Clinic, Instituto Nacional de Cardiología "Ignacio Chávez", Juan Badiano No.1, Colonia Sección XVI Tlalpan, 14080 México, D.F., Mexico

## Abstract

The aim of the present study was to describe the clinical and echocardiographic findings of ventricular noncompaction in adult patients. Fifty-three patients underwent complete clinical history, electrocardiogram, Holter and transthoracic echocardiogram. Forty patients (75%) were in class I/II of the New York Heart Association, and 13 (25%) in class III/IV. Ventricular and supraventricular escape beats were found in 40% and 26.4%, respectively. Holter showed premature ventricular contractions in 32% and sustained ventricular tachycardia in 7.5%. Ventricular noncompaction was an isolated finding in 74% of cases and was associated with other congenital heart disease in 26%. Noncompacted ventricular myocardium involved only left ventricle in 62% of the patients and both ventricles in 38%. The mean ratio of noncompacted to compacted myocardial layers at the site of maximal wall thickness was 3.4 ± 0.87 mm (range 2.2–7.5). The presence of ventricular noncompaction in more than three segments was associated with a functional class greater than II and ventricular arrhythmia with demonstrable statistical significance by χ^2^(p < 0.003).

**Conclusion:**

a) Noncompacted cardiomyopathy is a congenital pathological entity that can occur in isolated form or associated with other heart disease and often involves both ventricles. b) A ratio of noncompacted to compacted myocardium greater than 3 and involvement of three or more segments are indicators of poor prognosis. c) Since the clinical manifestations are not sufficient to establish diagnosis, echocardiography is the diagnostic tool that makes it possible to document ventricular noncompaction and establish prognostic factors.

## Background

Noncompaction of ventricular myocardium is recently included in the 2006 classsification of cardiomyopathies as a Genetic Cardiomyopathy [1,2]. Ventricular noncompaction occurs because of a disorder of endomyocardial morphogenesis that results in a failure of trabecular compaction of the developing myocardium [3-5]. Ventricular noncompaction is often associated with other congenital cardiac malformations [6-8]. In adult patients one or more segments of the left ventricle, and sometimes both ventricles, are characterized by numerous sinusoids or trabeculae that are excessive in number and abnormal in prominence and by deep intratrabecular recesses covered by endothelium that exhibits continuity with ventricular endocardium. Two-dimensional echocardiography provides definition of typical anatomic features. Jenni *et al*.[9] established four morphologic criteria for echocardiographic diagnosis that allow accurate differentiation from other forms of left ventricular hypertrophy. On the basis of echocardiographic studies, the prevalence of ventricular noncompaction has been estimated at 0.05% in the general population [3,10].

The aim of the present study was to describe the clinical and echocardiographic data of isolated and non-isolated ventricular noncompaction in adult patients from the Outpatient Clinic of the Instituto Nacional de Cardiología "Ignacio Chávez".

## Materials and methods

A total of 125,438 patients were studied in the period between May 2000 and August 2005 in our hospital and from these patients fifty-three had ventricular noncompaction. Patients were identified on their scheduled routine visits to the Outpatient Clinic and included 25 men and 28 women with an average age of 43.17 years (range 16 to 74 years). Isolated noncompacted cardiomyopathy was present in 39 (74%) and noncompacted cardiomyopathy associated with other congenital malformations of the heart in 14 (26%). All underwent complete clinical histories, 12 lead surface electrocardiogram and transthoracic M-mode, two-dimensional and Doppler echocardiograms. The clinical condition of each patient was noted until the end of the study or until the patient succumbed. Functional class was assessed according to the criteria of the New York Heart Association. Sudden death was defined as occurring within one hour of the patient's usual state of health or as unwitnessed death during sleep. Twenty-four hour electrocardiographic monitoring was performed on patients with histories of palpitations or cardiogenic syncope. Short runs of ventricular tachycardia were considered to be more than three premature ventricular contractions lasting up to 30 seconds. A ventricular run of more than 30 seconds was defined as sustained ventricular tachycardia [3,11].

### Echocardiography

A complete echocardiographic study was performed on all patients. Hewlett Packard Sonos 5500 equipment (HP, Andover, Massachusetts, USA.) was used. The echocardiographic diagnosis of noncompacted cardiomyopathy was established according to the criteria of Jenni *et al*.[9].

• The characteristic appearance of numerous, excessively prominent trabeculae and deep intratrabecular recesses observed in one or more ventricular wall segments;

• A maximum end-systolic ratio of noncompacted to compacted layers of > 2;

• Intertrabecular spaces filled by direct blood flow from the ventricular chamber, as visualized on color Doppler imaging.

Echocardiographic data were reviewed and interpreted by an experienced echocardiographer (NEZ) to confirm diagnosis.

Echocardiographic measurements included end-diastolic and end-systolic left ventricular diameters from parasternal long-axis images, left ventricular ejection fraction from apical four and two chamber images according to Simpson's method, and the ratio of noncompacted wall to compacted wall of both ventricles in end-systole from parasternal short-axis images. Color Doppler was used to establish the continuity of flow between the chamber and the intertrabecular recesses and to evaluate the distribution of the prominent trabeculae in the left ventricular using parasternal short axis and apical 4 and 2 chamber images. In 15 patients (28%) due to the poor acoustic window, an intravenous bolus of 1.0 mL of Definity contrast agent (Perfluoropropane, Manufacturer-Dupont) was administered over 1 second, followed by a flash of 0.9% sodium chloride, to enhance image quality. Parasternal short axis and apical 4 and 2 chamber views were used.

Diastolic function was evaluated from apical four chamber images with the sample volume placed at the tip of the mitral valve leaflets. Diastolic function was graded as normal, abnormal relaxation and restrictive pattern using previously described criteria [12].

The presence of intra and extracardiac shunts as well as valve stenosis and/or regurgitation were evaluated with color and continuous wave Doppler from parasternal long and short axis, suprasternal and apical 4 and 2 chamber images. Clinical follow-up was obtained on all patients based on notes from the last visit to Outpatient Clinic in the patients' charts or by telephone.

### Statistical analysis

Descriptive data for continuous variables were presented as mean ± one standard deviation. Chi-square analysis or Fisher exact tests were used for nominal data. Mann Whitney U or Student's t tests were used for quantitative data according to the case. Differences were considered significant when the p value was less than 0.05. Survival analysis for patients in functional class III/IV, with ventricular arrhythmia and ventricular noncompaction in three or more segments was determined by Kaplan Meier with the Log Rank test.

## Results

### Clinical and demographic characteristics

Fifty-three patients with a mean age of 43.2 ± 14.76 years (16–74) were studied. Twenty-five (4.2%) were men and 28 (42.8%) women. The prevalence of noncompacted cardiomyopathy in the Instituto Nacional de Cardiología "Ignacio Chávez" is of 4 per 10,000 patients per 5 years (Table 1).

**Table 1:** Demographic characteristics of the 53 patients

	**Number**	
**Male gender**	25	47.2%
**Age at Diagnosis, All**	43.2 ± 14.76	range 16–74
**Age at Diagnosis, Men**	40.9 ± 13.65	range 16–60
**Age at diagnosis, Women**	45.2 ± 15.65,	range 21–74
		
**Age, years**	**Number**	**Percent**
10–20	2	3.8
21–30	13	24.5
31–40	7	13.2
41–50	11	20.8
> 50	20	37.7
**Duration**	7 ± 5 months	range 1–24
**Prevalence in the INCICh**	4/10,000/5 years	

The primary diagnosis was missed in most cases. Incorrect diagnoses included dilated cardiomyopathy (n = 30), dilated phase hypertensive cardiomyopathy (n = 1), restrictive cardiomyopathy (n = 1), congenital heart disease (n = 6), ischemic heart disease (n = 2) and disease of the heart valves (n = 2). The primary diagnosis in the most recent eleven patients was ventricular noncompaction. Several echocardiographic studies were required to establish the diagnosis of ventricular noncompaction in most of the cases.

Forty patients (75%) were in class I/II of the New York Heart Association (NYHA); 13 (25%) were in NYHA class III/IV. Eleven patients (21%) had chest pain and 5 (9.4%) syncopal events. The surface ECG revealed sinus rhythm in 90.5% of the cases. Ventricular and supraventricular premature contractions were observed in 40% and 26.4%, respectively.

Twenty-four hour ambulatory ECGs showed short runs of premature ventricular contractions in 32% of the patients and sustained ventricular tachycardia in 7.5%.

Sixteen patients (30.2%) had family histories of ventricular noncompaction (Table 2).

**Table 2:** Clinical and electrocardiographic characteristics

**Finding**	**Number**	**Percent**
**Precordial pain**	11	21
**Syncope**	5	9.4
**NYHA Functional class I/II**	40	75
**NYHA Functional class III/IV**	13	25
**Familiar occurrence**	16	30.2
***Cardiac Rhythm***		
Sinus	48	90.5
Atrial fibrillation	3	5.7
Pacemaker	2	3.8
Bundle branch block	25	47.2
Left	18	72
Right	7	28
With premature ventricular contractions	21	40
With supraventricular escape beats	14	26.4
***24 hour Holter***		
Runs of premature ventricular contractions	17	32
Sustained ventricular tachycardia	4	7.5

### Echocardiographic findings

Echocardiographic data are shown in Table 3. The left ventricular end-diastolic diameter was 58 mm ± 11.38 (34–87), the end-systolic diameter was 45 mm ± 13.35 (21–69) and the left ventricular ejection fraction was 39% ± 18.5 (15–75).

**Table 3:** Echocardiographic findings

LVEDD	58 ± 11.38	(normal: < 50 mm)
LVESD	45 ± 13.35	(normal: <33 mm)
Left ventricular ejection fraction	39 ± 18.5	(normal ≥ 50%)
Dp/Dt (n = 38)	535 ± 194.7	(normal: > 1000)
		
***Diastolic function***		
Impaired relaxation	14 (26.4%)	(E/A < 1.0)
Restrictive pattern	26 (49.1%)	(E/A ≥ 1.5)
Normal	13 (24.5%)	(E/A = 1.0–1.49)
**Thrombus**	3 (5.7%)	
Left ventricle	2	
Left atrium	1	
**Pericardial effusion**	3 (5.7%)	
		
***Valvular regurgitation***		
Mild mitral	15 (28%)	
Moderate-Severe mitral	23 (43%)	
Moderate aortic	1 (1.9%)	
Mild tricuspid	17 (32%)	
Moderate-Severe tricuspid	17 (32%)	
**Isolated ventricular noncompaction**	39 (74%)	
**Ventricular noncompaction associated with other congenital anomalies**	14 (26%)	
		
***Localization of ventricular noncompaction***		
Left ventricle	33 (62%)	
Both ventricles	20 (38%)	
**Ratio of Noncompacted to Compacted Wall**	3.4 ± 0.87	

**LVEDD**: Left ventricular end-diastolic diameter; **LVESD**: Left ventricular end-systolic volume

Diastolic function was evaluated in 48 patients (90.5%). Thrombi were found in the left ventricles of 2 patients and in the left atrium in one. Two of these patients developed cerebral infarctions.

Three patients (5.7%) presented pericardial effusions.

Moderate to severe mitral regurgitation was detected in 43% of the patients, aortic regurgitation in 1.9% and moderate to severe tricuspid regurgitation in 32%.

**Figure 1:**
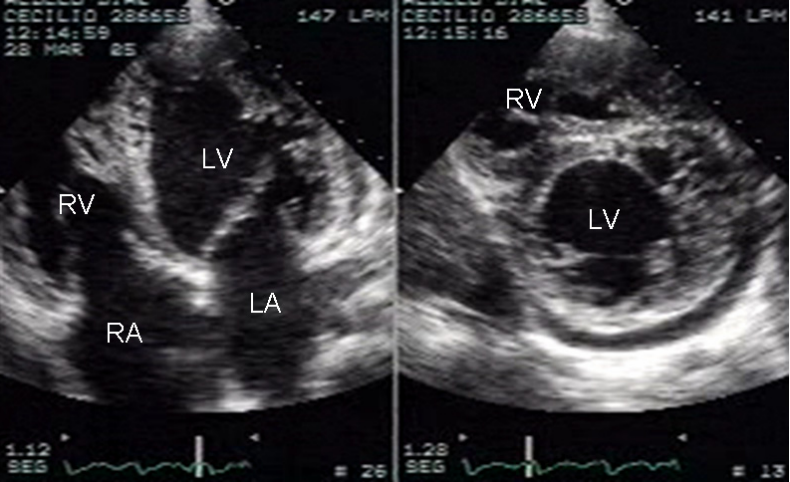
Two-dimensional apical four chamber and parasternal short axis images at the level of the ventricles show dilatation of both ventricles, multiple trabeculae and intertrabecular recesses in inferior, lateral, anterior walls, middle and apical portions of the septum and apex of the left ventricle. A mild pericardial effusion can be observed. LV: Left ventricle; LA: Left atrium; RV: Right ventricle; RA: Right atrium.

**Figure 2:**
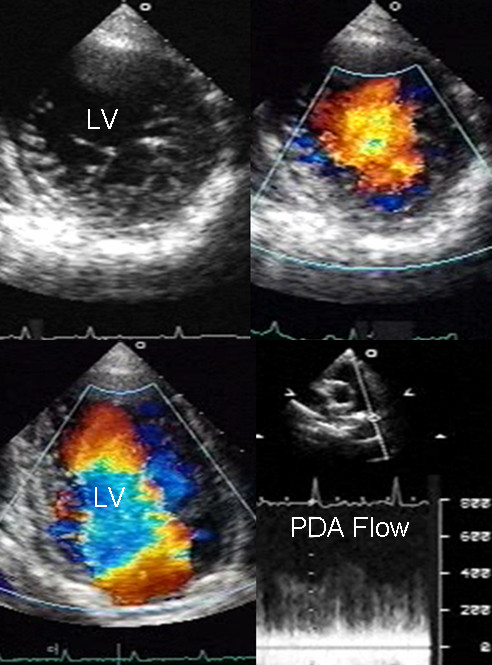
Transthoracic two-dimensional study with color and continuous wave Doppler shows left ventricular noncompaction associated with patent ductus arteriosus (PDA). Trabeculae and deep recesses with penetration of color can be observed in the left ventricle. Continuous wave Doppler from a suprasternal approach at the level of the great vessels registers systolic-diastolic flow through the ductus arteriosus. Others abbreviations as before.

**Figure 3:**
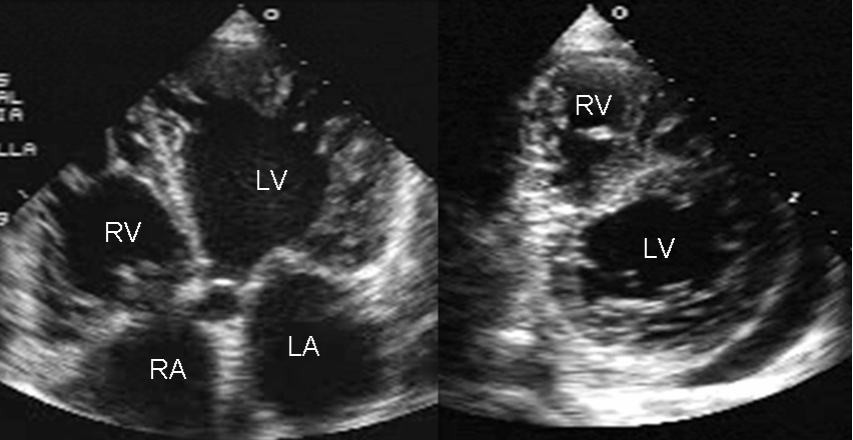
Transthoracic two-dimensional echocardiogram in apical four chamber and parasternal short axis at the level of both ventricles demonstrate dilatation, deep trabeculae and intertrabecular recesses in the inferior, lateral, anterior walls, middle and apical portions of the septum and apex of the left ventricle. The right ventricle also shows evidence of noncompaction. A posterolateral pericardial effusion is also present. Others abbreviations as before.

Ventricular noncompaction was an isolated finding in 74% of the cases (Figure 1) and was associated with other congenital abnormalities of the heart in 26% (Figure 2, Table 4). In 62% of patients noncompacted ventricular myocardium involved only the left ventricle and in 38% both ventricles (Figure 3).

**Table 4:** Congenital heart disease associated with ventricular noncompaction n = 14

Ebstein's Anomaly + ASD	2
Uhl's Anomaly + ASD	1
Atrial septal aneurysm + PFO	2
Double outlet right ventricle + Pulmonary stenosis	1
Asymmetric septal hypertrophic cardiomyopathy	1
Unicuspid mitral valve	2
Atrial septal defect + MVP	1
Double mitral orifice + Moderate mitral regurgitation	1
Bicuspid aortic valve + Moderate aortic regurgitation	1
Persistent ductus arteriosus	2

ASD = Atrial septal defect, PFO = Patent foramen ovale

**Figure 4:**
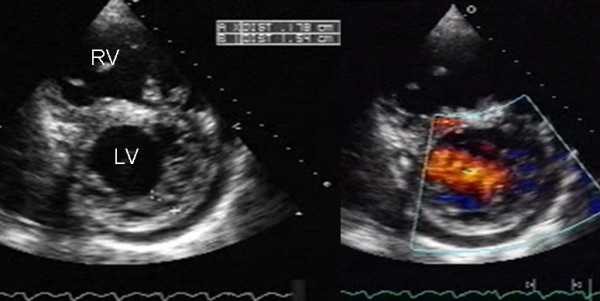
Two-dimensional parasternal and color Doppler images at the level of both ventricles that show the noncompacted:compacted wall ratio and how the color enters the intertrabecular recesses. Others abbreviations as before.

The ratio of noncompacted to compacted myocardial layers at the site of maximal wall thickness averaged 3.4 ± 0.87 mm (range 2.20–7.5). Color Doppler analysis showed typical forward and reversed directional blood flow from the ventricular chamber into the spaces between the prominent trabeculae (Figure 4).

**Figure 5:**
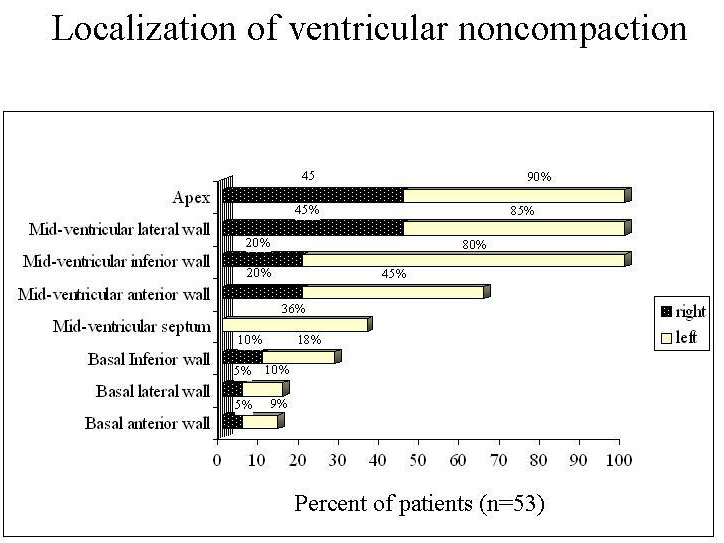
Graph shows the percentage of different segments of the left and right ventricular wall affected by noncompaction.

Localization of noncompacted myocardial segments is shown in Figure 5. Three or more segments were involved in 42 (80%) patients. All noncompacted segments were hypokinetic.

The ratio of noncompacted to compacted myocardium in patients in functional class III/IV was significantly greater, with an average of 4.2 ± 1.2 (range 3.0–7.5), when compared to patients in functional class I/II, in whom the average ratio was 3.2 ± 0.61 (range 2.2–5.0) (p < 0.05).

The localization of ventricular noncompaction in more than three segments was more frequently associated with a functional class greater than II and ventricular arrhythmia than in patients with fewer affected segments. The difference was statistically significant (p < 0.003).

A total of 12 patients (23%) were found to manifest ventricular arrhythmia, ventricular noncompaction in three or more segments and functional class III/IV.

### Follow-up data

The mean follow-up was 7 months ± 5 (range 1–24). Major complications are presented in Table 5. Heart failure requiring hospitalization (13%) was the most frequent event.

**Table 5:** Patient Follow-up

Heart failure requiring hospitalization	7 (13%)
Deaths	3 (5.7%)
Heart failure	3
	
Implantation of intracardiac defibrillator	3 (5.7%)
	
Thromboembolic events	3 (5.7%)
Cerebrovascular accident	2
Transient ischemic attack	1

Three (25%) of the 12 patients with noncompacted myocardium in three or more segments, in functional class III/IV and ventricular arrhythmias succumbed.

The 10 patients in functional class III/IV who survived received complete medical treatment for heart failure (digitalis, ACE inhibitors, diuretic, beta blockers and antiplatelet agents and/or anticoagulants). Of these, three patients had defibrillators implanted to improve symptoms of heart failure refractory to complete medical treatment with favorable results.

Three patients (5.7%) presented thromboembolic events, including two cerebral infarctions and on transitory cerebral ischemia.

## Discussion

**Figure 6:**
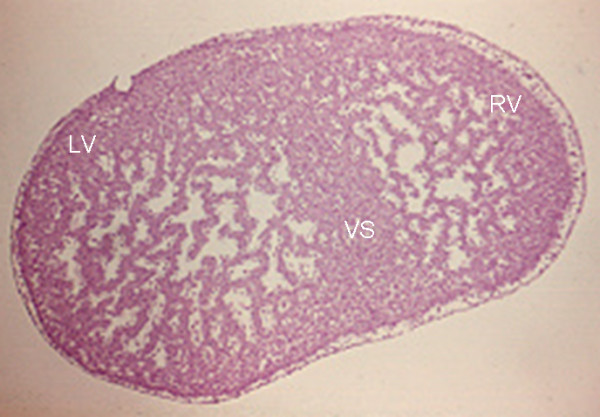
Microphotograph of a transverse section at the level of both ventricles of a heart that shows extensively developed trabeculae that fill the ventricular lumen. Note the form of the more compacted ventricular septum (arrow). 250 X. VS-Ventricular septum. Others abbreviations as before.

In the human embryo of Streeter's horizon [Streeter's horizons are stages of early human embryonic development, which establish criteria of external form and internal structure that characterize each group ("horizon")] XII appear trabeculated or sinusoidal pouches in both cardiac ventricles [13]. One is in the bulbis cordis (right ventricle) and the other in the primitive ventricle (left ventricle). The proliferation of the myocardial trabeculae covered with endothelium and a thin layer of cardiac gelatin diminishes the central lumen of the ventricles during their centrifugal growth. In horizon XVII [14,15] the trabeculae have extended from the apical to the inlet portions, leaving only the outflow tracts smooth. Because of this the ventricles have a non-compacted spongy nature (Figure 6). The cardiac chambers undergo compaction as the trabeculae fuse with each other and with the ventricular walls. This process is very advanced in Streeter's horizon XVIII [14]. The first reported cases of ventricular noncompaction were associated with such congenital malformations as obstruction of the left and right ventricular outflow tracts, complex congenital malformations and coronary anomalies [16].

Isolated noncompaction of the ventricular myocardium was first reported by Chin in 1990 [17]. In these cases the sinusoids are open to the ventricular chamber but do not communicate with the coronary circulation. In adults familial recurrence ranges between 18% and 50% [18]; in our series familial recurrence was 30.2%.

Noncompacted myocardium can be considered an inherited congenital alformation since the genes responsible for its development have been identified on chromosome 11p15 [19] or as mutations of the gene 4.5 of chromosome Xq28, where other cardiomyopathies have been identified [20]. New mutations of gene 4.5 have been reported, and mutations of the alpha-distrobrevine gene have been found in patients with ventricular noncompaction associated with other congenital malformations of the heart [16]. Mutation of the FKBP12 gene produces ventricular septal defects, dilated cardiomyopathy and noncompacted cardiomyopathy [21]. The CSX gene has been implicated in the development of some cases of isolated noncompacted cardiomyopathy [22].

The clinical manifestations of noncompacted cardiomyopathy are variable. Patients may be asymptomatic or may demonstrate evidence of congestive heart failure, arrhythmias or systemic emboli [11,21,23,24], as seen in our series.

The echocardiogram is the diagnostic procedure of choice, and diagnosis is based on established criteria [9]. Contrast-enhanced echocardiography has recently emerged as a noninvasive tool for better visualization of the endocardial blood-interface [25]. Its use in the diagnosis of ventricular noncompaction should be recommended, especially in sub-optimal studies, because allows a better delineation of the trabeculae and deep intratrabecular recesses, also the intertrabecular spaces filled by microbubbles is clearly observed.

However, diagnosis is sometimes overlooked or delayed because this disease is rare and not well-known [11]. This occurred in 79% of our patients, who were initially diagnosed with other conditions. In our institution the prevalence of noncompacted cardiomyopathy was 4/10,000/5years. While 77% of our patients were in functional class I/II, the remaining 33% manifested frank congestive heart failure. Ventricular arrhythmias (premature ventricular contractions and short runs of ventricular tachycardia) were documented in more than a third of the patients. Precordial pain, syncope and cerebral embolism also occurred less frequently.

**Figure 7:**
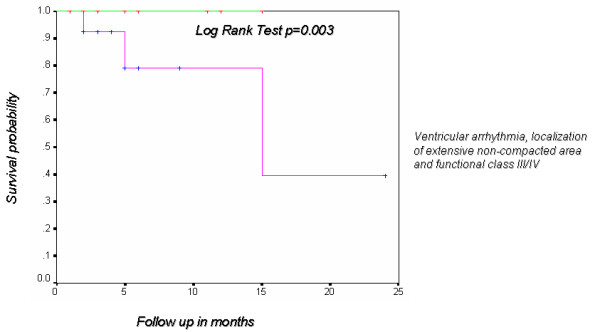
Kaplan Meier curve that shows the probability of survival in patients with ventricular arrhythmia, localization of extensive non-compacted area and functional class III/IV.

Ventricular noncompaction involving three or more segments was found in 80% of the cases. Left ventricular apical, inferior and lateral walls were predominantly affected. Ventricular noncompaction was initially reported only in middle and apical portions of the left ventricle. However, in a minority of patients it also affected basal segments. A finding relevant to our series is that patients with a noncompacted:compacted ratio greater than 3 and ventricular noncompaction in three or more segments are in functional class III/IV rather than functional class I/II. For patients in functional class III/IV with ventricular arrhythmia and noncompaction of three or more segments, probability of survival to 9 months was 75%. Probability of survival to 15 months in these patients was 48% (Figure 7).

In 26% of our patients noncompacted cardiomyopathy was associated with other congenital malformations of the heart, some of which have been reported previously [6-8], while others such as Uhl's anomaly, atrial septal defects and right ventricular double outlet, have not been described in association with ventricular noncompaction.

Noncompacted ventricular myocardium typically involves one or more segments of the left ventricle. The right ventricular apex is often intensely trabecular, which makes it difficult to distinguish normal and pathologic patterns. However, prominent trabeculae and hypokinesis of right ventricular wall accompanied by left ventricular noncompaction permits diagnosis of right ventricular involvement [6]. In Ritter's series of noncompacted cardiomyopathy, the right ventricle was affected in 41% of patients with concomitant left ventricular noncompaction [3]. We found noncompaction of both ventricles in 38% of the patients in our series.

On the basis of our findings we may conclude that:

a) Noncompacted cardiomyopathy is a congenital malformation that can occur as an isolated entity or associated with other pathologies of the heart and can often involve both ventricles.

b) A ratio of noncompacted:compacted wall greater than 3 and involvement of three or more segments are signs of poor prognosis associated with greater clinical deterioration (functional class III/IV) and ventricular arrhythmias.

c) Inasmuch as the clinical picture does not provide sufficiently specific evidence to establish the diagnosis, the echocardiogram is the diagnostic cornerstone. It makes it possible to document noncompacted ventricular myocardium and to identify the factors of poor prognosis.
